# Elevated Frequency of Cataracts in Birds from Chernobyl

**DOI:** 10.1371/journal.pone.0066939

**Published:** 2013-07-30

**Authors:** Timothy Alexander Mousseau, Anders Pape Møller

**Affiliations:** 1 Department of Biological Sciences, University of South Carolina, Columbia, South Carolina, United States of America; 2 Laboratoire d'Ecologie, Systématique et Evolution, CNRS UMR 8079, Université Paris-Sud, Orsay, France; Estacion Experimental de Zonas Áridas (CSIC), Spain

## Abstract

**Background:**

Radiation cataracts develop as a consequence of the effects of ionizing radiation on the development of the lens of the eye with an opaque lens reducing or eliminating the ability to see. Therefore, we would expect cataracts to be associated with reduced fitness in free-living animals.

**Methodology/Principal Findings:**

We investigated the incidence of lens opacities typical of cataracts in more than 1100 free-living birds in the Chernobyl region in relation to background radiation. The incidence of cataracts increased with level of background radiation both in analyses based on a dichotomous score and in analyses of continuous scores of intensity of cataracts. The odds ratio per unit change in the regressor was 0.722 (95% CI 0.648, 0.804), which was less than odds ratios from investigations of radiation cataracts in humans. The relatively small odds ratio may be due to increased mortality in birds with cataracts. We found a stronger negative relationship between bird abundance and background radiation when the frequency of cataracts was higher, but also a direct effect of radiation on abundance, suggesting that radiation indirectly affects abundance negatively through an increase in the frequency of cataracts in bird populations, but also through direct effects of radiation on other diseases, food abundance and interactions with other species. There was no increase in incidence of cataracts with increasing age, suggesting that yearlings and older individuals were similarly affected as is typical of radiation cataract.

**Conclusions/Significance:**

These findings suggest that cataracts are an under-estimated cause of morbidity in free-living birds and, by inference, other vertebrates in areas contaminated with radioactive materials.

## Introduction

Cataract is a disease of the eye characterized by an opaque lens that under normal conditions focus images on the retina to allow perception of visual information. Such opacities result in a reduction in vision and even complete loss of eyesight, and cataracts are the single-most common cause of blindness in humans. The lens of the eye has for a long time been considered sensitive to ionizing and non-ionizing radiation [1], [2]. Cataracts are a frequent outcome of exposure to radiation [e. g. 3]–[8]. The underlying mechanism by which ionizing radiation causes cataracts is the ionization of water and the production of free radicals such as hydroxyl and hydrogen radicals. These free radicals can damage DNA and cause damage or errors in lens protein formation and hence contribute to cataract formation. The lenses are almost entirely composed of proteins that can be oxidized with subsequent aggregation and precipitation causing opacities in the lenses that form the basis of cataracts. Lens proteins show little turnover throughout the life of an individual thereby making them particularly susceptible to oxidative stress. Dose thresholds of 0.5–2.0 Gy for acute exposure and 5.0 Gy for protracted exposure for radiation cataract are assumed in the literature [9]. However, to date the most extensive epidemiological study of 8607 Chernobyl Ukrainian liquidators involved in clean-up at the Chernobyl nuclear power plant showed a post-exposure level of dose of only 0.12 Gy, and despite the low level of dose there was a statistically significant dose response for stage 1 cataract (the initial stage of cataracts) and posterior subcapsular cataracts (located in the subcapsular region of the ocular lens) [7]. Threshold exposure estimates showed that values greater than 700 mGy caused a significant risk of detectable opacities, which is a much greater risk than what is recommended in radiation protection guidelines [10]. Studies of atomic bomb survivors have shown a clear linear dose-response for the frequency of cataracts without a threshold and this is much lower than the threshold of 2–5 Gy usually assumed by the radiation protection community [11]. A similar conclusion was reached for a study of radiological technologists [12]. Thus there is evidence consistent with low or no thresholds [13], and this documented greater susceptibility to radiation than proposed by radiation protection authorities is consistent with a recent assessment showing that to generally be the case [14].

The eye lens is almost entirely composed of proteins whose oxidation and subsequent aggregation and precipitation generate the opacities characteristic of cataracts [15]. Some cases of cataracts in humans have a genetic basis [16], although the main causes of human cataracts are agents that produce oxidative stress (i.e., the imbalance between levels of reactive oxygen species and state of the antioxidant and repair machinery) such as ultraviolet radiation and smoking, as well as accumulation of effects of free radicals with aging [15], [17]. Accordingly, Galván et al. [18] showed elevated levels of cataracts in wild birds with pheomelanin-based plumage coloration, apparently linked to the effects of oxidative stress. Cataracts are a rare phenomenon in wild animals [19] because any deterioration in vision will likely very soon be followed by death due to predation or lack of ability to find adequate and sufficient food for survival.

The objectives of this study were to determine (1) the relationship between the incidence of cataract and background radiation, using a large database of more than 1100 free-living birds captured at Chernobyl during 2011–2012. In other words, we assessed the reliability of the rate of cataracts as a biomarker of radiation exposure. Because the lifespan of most free-living animals with impaired vision is bound to be short due to elevated risk of predation and inability to find sufficient amounts of food, field estimates of prevalence of cataracts are by definition conservative. Therefore, the second objective of this study was (2) to determine whether the abundance of different species of birds decreased the most in species that showed a stronger impact of radiation on the incidence of cataracts. Finally, we tested (3) if the frequency of cataracts increased with age as expected from human studies showing accumulation of free radicals with age [3]–[8]. Because many birds can be aged reliably as either yearlings or older individuals [20], we included a categorical age variable and the interaction between level of background radiation and age as predictors of the extent of cataracts.

## Methods

### Ethics Statement

The research complied with requirements for research on birds in Ukraine, and permission was given by the administration of the Chernobyl Excclusion Zone. All sampling was approved in an ethical review by the University of South Carolina Institutional Animal Care and Use Committee. All birds were handled shortly, and none died or showed signs of suffering during the short examination. All individuals flew upon release.

### Study Areas

**Figure 1:**
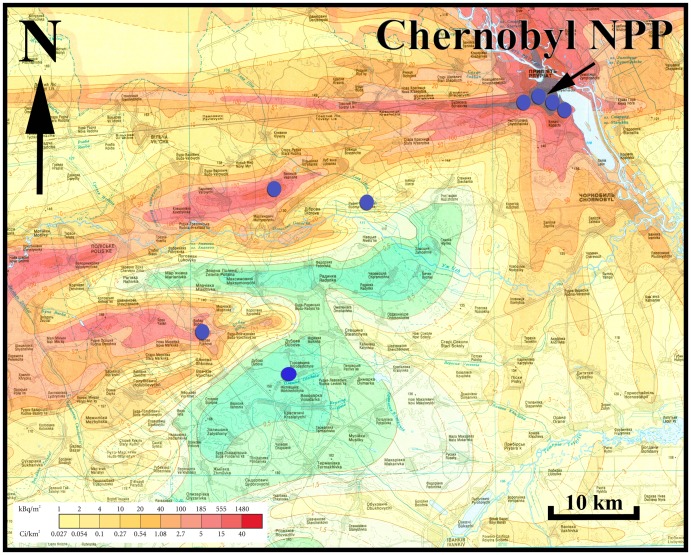
Location of the eight study sites in relation to background radiation level (Ci/km^2^) around Chernobyl, Ukraine. Adapted from Shestopalov (1996).

The research complied with requirements for research on birds in Ukraine, and permission was given by the administration of the Chernobyl Exclusion Zone. All birds were handled shortly, and none died or showed signs of suffering during the short examination. All individuals flew upon release. We captured birds in mist nets at eight sites within and just outside the Chernobyl Exclusion Zone on 25 May–5 June 2011–2012 from four pairs of relatively uncontaminated and contaminated sites (Figure 1). We used 35–45 mist nets each 12 m long for two consecutive days at each of the study sites (i.e. one evening and one morning capture session at each site). All birds were banded with a unique aluminum band for individual identification, and they were subsequently sexed and aged according to standard criteria [20], measured, sampled for blood and sperm, and released.

### Frequency of Cataracts

**Figure 2:**
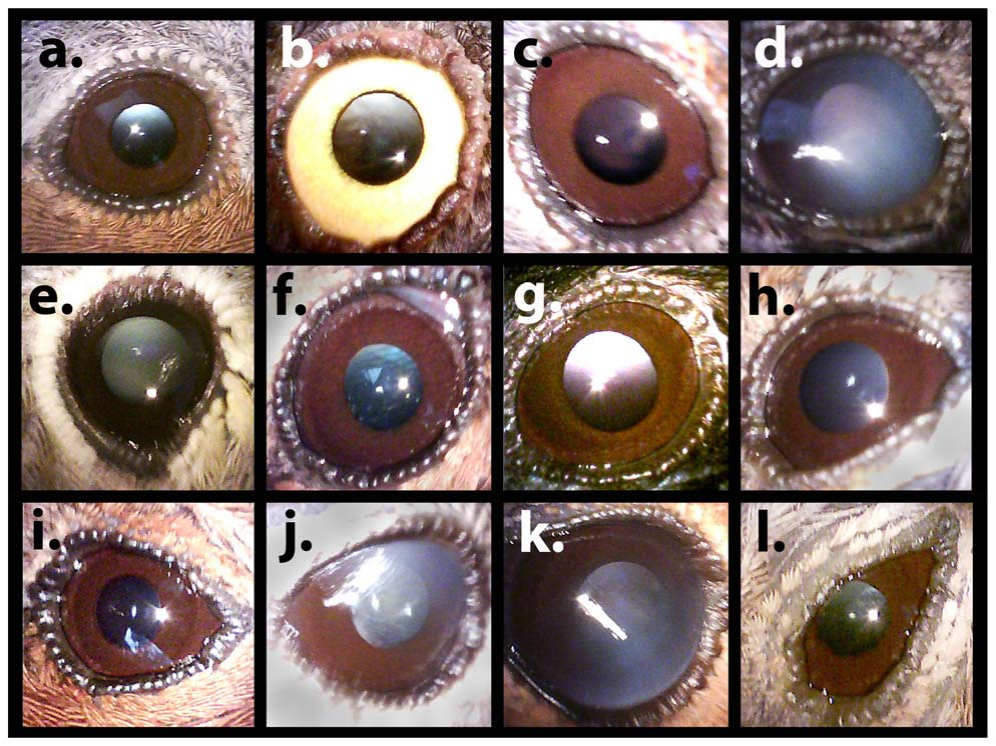
Photographs of selected eyes from Chernobyl birds. (a) Blackcap (*Sylvia atricapilla*), normal – score 0; (b) barred warbler (*Sylvia nisoria*), normal – score 0; (c) blackcap (*Sylvia atricapilla*), slight haze in cornea – score 1; (d) barn swallow (*Hirundo rustica*), significant haze on cornea – score 2; (e) chiffchaff (*Phylloscopus collybita*), slight haze on cornea – score 1; (f) chiffchaff (*Phylloscopus collybita*), significant haze on cornea – score 1; (g) barn swallow (*Hirundo rustica*), significant haze on cornea – score 3; (h) chaffinch (*Fringilla coelebs*), slight haze on cornea – score 1; (i) chaffinch (*Fringilla coelebs*), clear eye but deformed eye lids – score 0; (j) tree pipit (*Anthus trivialis*), significant opacity of cornea – score 2; (k) robin (*Erithacus rubecula*), significant haze on cornea – score; (l) chiffchaff (*Phylloscopus collybita*), deformed eye lids, haze on cornea – score 2. All photos captured using an EyeQuick Digital Ophthalmoscope Camera.

Upon capture all individuals were examined by TAM for the presence of cataracts or minor opacities in the eyes. This was done without prior knowledge of the level of background radiation, thereby making the assessment blind with respect to radiation levels. Eyes were visually inspected using a standard medical ophthalmoscope (model ADC 5215) usually at 40× magnification although some larger birds (e.g. hawfinches *Coccothraustes coccothraustes*) were inspected at 20× magnification because of the larger eye and the need for longer examination distances. Examinations were conducted under a black, lightproof hood to induce iris dilation and reduce interference from background lighting. Each eye was scored to the nearest 0.5 on a scale from 0 to 4 with 0 indicating total clarity and scores 1, 2, 3 and 4 denoting increasing extent of opacity reaching complete opacity at a score of 4. Such a discrete five-point scale is similar to what has been used in studies of radiation cataracts in humans [7]. There was considerable variation among individuals in opacification (Fig. 2) with most eyes falling between 1 and 2. A lens was scored as 1 if there was any suggestion of cloudiness in the cornea or lens, 2 if the cloudiness extended across most of the cornea or lens, and 3 if the cloudiness was sufficiently opaque to obscure details in the iris. A score of 4 indicated the presence of a partial or complete cataract. Occasionally birds, most noticeably smaller species, would close one or both their eyes in response to being placed inside the darkened environment, preventing inspection of one or both eyes for such individuals. In this case we used the score for the open eye as an estimate of the mean cataract score for the two eyes. Although this method requires a period of familiarization and internal calibration for the observer, scores were highly repeatable when observations of the same individuals were taken independently by TAM and APM (cataract score: *R = *0.93 (SE = 0.04), *F* = 27.56, d.f. = 19, 20, *r* ^2^ = 0.96, *P*<0.0001; dichotomous score: *R = *0.81 (SE = 0.11), *F* = 9.47, d.f. = 19, 20, *r* ^2^ = 0.90, *P*<0.0001), as has been reported for similar approaches used for human subjects [21]. The overall procedure lasted 1–2 minutes per bird and was similar in all study sites.

A selection of photos of the eye is provided in Figure 1. All photos were captured using an EyeQuick Digital Ophthalmoscope camera on loan from EyeQuick, LLC. Unfortunately, because of the constraints of working under field conditions and the operator's insufficient training, it was not possible to consistently and reliably capture images from all birds using this technique. The images presented here serve only to illustrate the range of variation in eye shape and opacity and cannot at present be used for quantitative analyses of variability.

### Breeding Bird Censuses

We assessed the relationship between abundance and radiation during the breeding season using point census counts. The point count census method provides reliable information on relative abundance of birds [22]–[25]. It consists of counts lasting 5 minutes during which the number of birds seen or heard was recorded with the next census point being located at a distance of more than 100 m. APM conducted these standard point counts during May–June 2006–2009 in sites around Chernobyl. The fact that one person made all counts eliminates any variance in results due to inter-observer variability. The counts were made approximately the same dates and at the same time of the day each of the four years. Møller & Mousseau [24] provide additional information on the methods and potentially confounding variables that were taken into account in the analyses. We have previously tested the reliability of our counts by letting two persons independently perform counts, and the degree of consistency was high for both species richness and abundance (details reported by Møller & Mousseau [24]). Relationships between abundance and radiation are repeatable among years [26]. We provide summary statistics for the frequency of cataracts in each species and the slope of the relationship between abundance and background radiation level in Table S1.

### Background Radiation

We measured the level of background radiation in the field and cross-validated these measurements with those reported by the Ukrainian Ministry of Emergencies. Once having finished the 5 minute point count we measured radiation levels at ground level directly in the field at each point where we censused birds using a hand-held dosimeter (Model: Inspector, SE International, Inc., Summertown, TN, USA). Likewise, we measured radiation at the exact capture spot for each individual bird. This was done 2–3 times at each site and the measurements were averaged. We have captured more than 1200 individual birds in the surroundings of Chernobyl during 2010–2012, and the radiation levels at which individuals were recaptured the same breeding season, but also the following season were highly repeatable, suggesting that a single measure of background radiation provide a reliable estimate of future exposure (T. A. Mousseau et al. unpublished data). We cross-validated our measurements against governmental measurements published by Shestopalov [27], estimated as the mid-point of the ranges published in the Chernobyl atlas. This analysis revealed a very strong positive relationship (linear regression on log-log transformed data: *F* = 1546.49, d.f. = 1, 252, *r* ^2^ = 0.86, *P*<0.0001, slope (SE) = 1.28 (0.10)), suggesting that our field estimates of radiation provided reliable measurements of levels of radiation among sites.

In Chernobyl, cesium-137, strontium-90, various isotopes of plutonium, and americium-241 are found at biologically significant levels across the landscape [28]. Cesium-137, with a half-life of about 30 years, decays by beta emission primarily to a meta-stable isomer of barium-137, which is responsible for the gamma emissions of this isotope [29]. Thus if ingested, cesium-137 will generate both beta and gamma doses for living organisms. Cesium-134 that has a half-life of about 2 years is exclusively a beta emitter and is thus mainly a concern if ingested. Strontium-90, with a half-life of about 29 years, is almost a pure beta emitter [29]. Most isotopes of plutonium are alpha emitters and are thus primarily of concern if ingested, due to the short travel distance of alpha particles. However, plutonium-241, which is present to a significant degree in the Chernobyl region, has a half-life of about 14 years, and decays via beta emissions to americium-241 (half life of 432 years), which in turn decays via alpha emissions to neptunium-237, with gamma emissions as an additional by-product.

Handheld Geiger counters provide reliable measures of background contamination levels of radionuclides for gamma sources, and to a lesser degree for beta emitters if the Geiger detector is in close proximity to the source. However, characterization of alpha emitters usually requires more complex measurement methods that are usually only tractable in a laboratory setting due to the short transmission distance of alpha particles in air.

Given the different characteristics of radionuclides in the environment at Chernobyl, field measurements of contaminant levels are likely to underestimate biologically relevant radiation levels when the main exposure pathway is via ingestion. Similarly, background radiation measures in the areas of Chernobyl closest to the reactor (e.g. the Red Forest) very likely underestimate biologically relevant doses given the abundance of alpha emitting actinides (e.g. plutonium isotopes) that were differentially deposited in this area. That said, Gashchak et al. [30] have previously reported that internal dose of small birds captured in Chernobyl is strongly positively correlated with external dose, accounting for more than 50% of the variance in internal dose. This result suggests that gamma radiation measurements are a reasonable proxy for total exposure at least for the sorts of comparative analyses reported here, and that results will be conservative with respect to any reported associations with radiation levels.

### Statistical Analyses

We quantified the incidence and the score of intensity of cataracts using the mist netted sample of birds. As a way of testing for consistency in cataract scores, we calculated the rank order correlation between cataract score for the left and the right eye.

We assessed the relationship between cataracts and background radiation in three different ways. First, we related our cataract score of all individuals to background radiation entering species as a random factor to account for the non-independence of multiple observations of different species. Second, we used nominal logistic regression to relate the incidence of cataracts to background radiation level after inclusion of species as a factor. Third, we used ordinal logistic regression to relate the incidence of cataracts (an ordinal variable) to background radiation level after inclusion of species as a factor. These three models showed no significant lack of fit. We subsequently related the slope of the relationship between abundance and background radiation to the score of cataracts in different species of birds, weighting each species estimate by sample size. Using GLM, we previously estimated the slope of the relationship between abundance and background radiation using log_10_-transformed abundance for each species at each observation point and log_10_-transformed radiation level for each species [26]. Models were weighted by sample size (the number of individuals assessed for cataracts) to account for the fact that sample sizes differed and hence the precision of the estimate differed among species. Most statistical analyses assume that data points provide equally precise information about the deterministic part of total process variation, i.e. the standard deviation of the error term is constant over all values of the predictor variable [31]. Garamszegi & Møller [32] showed that bias due to variation in sample size is a major problem in comparative analyses. If this assumption of even sampling effort is violated, weighting each observation by sampling effort allows the use of all data, giving each datum a weight that reflects its degree of precision due to sampling effort [31], [33], [34]. This procedure also allows both rare and common species to be included and hence avoids any bias in sampling due to rarity [35]. All analyses were made with JMP version 10.0 [36].

We tested if there were phylogenetic patterns in the cataracts data by means of nested analyses with species nested within genus, genera nested within families and families nested within orders.

We performed a path analysis [38] of the relationship between background radiation, cataracts and population trends in relation to background radiation. Basically, we estimated the partial regression coefficients for the paths between background radiation, cataracts and abundance.

We estimated effect sizes by relying on Cohen's [37] guidelines for the magnitude of effects, proposing explicit criteria for judging whether effects are small (Pearson *r* = 0.10, explaining 1% of the variance), intermediate (*r* = 0.30, explaining 9% of the variance) or large (*r* = 0.50, explaining 25% of the variance).

## Results

There was a highly significant Kendall rank order correlation between cataract scores for the two eyes (Kendall τ = 0.48, *P*<0.0001), this score was significantly repeatable (*R = *0.72 (SE = 0.02), *F = *6.09, d.f. = 1077, 1144, *r* ^2^ = 0.85, *P*<0.0001), and, we used the mean score in subsequent analyses. In Chernobyl, 391 out of 1111 individual birds or 35.2% had a cataract score of 1 or more.

**Figure 3:**
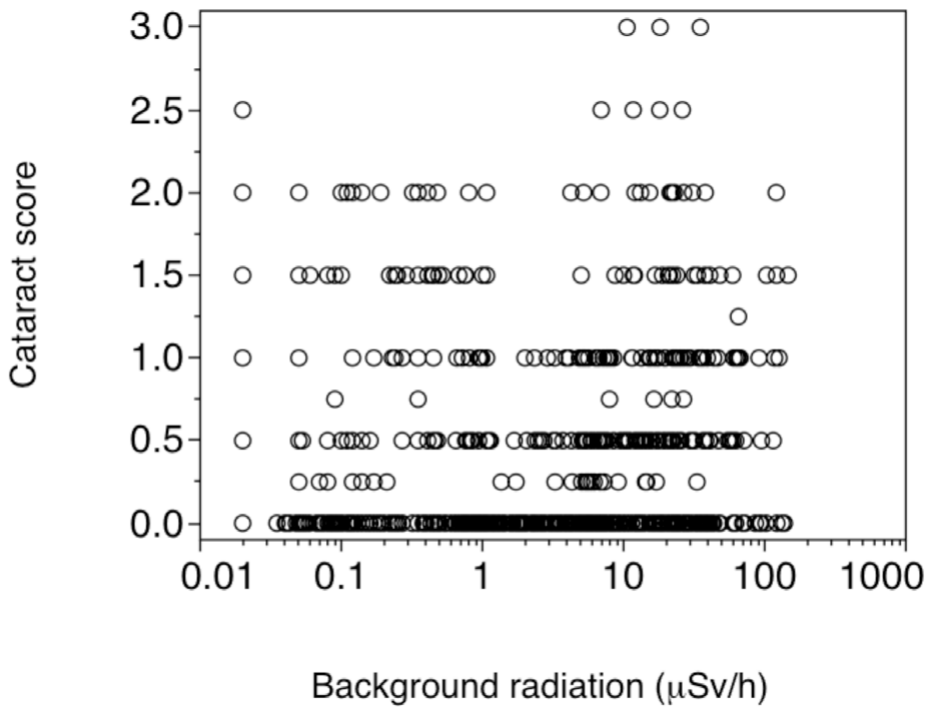
Mean cataracts in birds from Chernobyl in relation to background radiation level (μSv/h). The linear fit with confidence intervals is shown.

A mixed model with mean cataract score as the response variable, species as a random effect and radiation as a continuous predictor accounted for 13% of the variance (Table 1; Figure 3). However, the residuals from this model were not normally distributed (Shapiro-Wilk W test, *W = *0.88, *P*<0.0001). The random species effect accounted for 7% of the variance with the rest attributed to radiation. A second model that used cataract score for both eyes as the response variable and background radiation and individual as a random factor likewise showed an effect of radiation (*F = *6.31, d.f. = 1, 1108, *P* = 0.012, estimate (SE) = 0.038 (0.015)) and species (variance ratio = 0.92, 95% CI = 0.054, 0.274). A third model based on logistic regression was significant (Χ^2 = ^232.75, d.f. = 57, *P*<0.0001) with no significant lack of fit (Χ^2^ = 1822.53, d.f. = 6630, *P* = 1.00). The effect of species was significant (Χ^2 = ^204.52, d.f. = 56, *P*<0.0001) as was the effect of radiation (Χ^2^ = 101.77, d.f. = 1, *P*<0.0001). Finally, a Kendall rank order correlation between cataract scores and background radiation was also significant (Kendall τ = 0.13, *P*<0.0001). When we added age to the random effects model, the partial effect of age was small and far from significant (*F = *0.19, d.f. = 1, 382, *P* = 0.66), and the same applied to the radiation by age interaction (*F = *0.0007, d.f. = 1, 382, *P* = 0.97). Thus all analyses supported a hypothetical effect of radiation on the frequency of cataracts independent of species effects.

**Table 1:** Mixed model of cataracts in relation to species (random factor) and radiation.

Variable	d.f.	*F*	*P*	Estimate (SE)
Intercept	48.66, 1074		<0.0001	
log Radiation	1, 1074	89.63	<0.0001	0.131 (0.014)

The random species effect accounted for a variance ratio of 0.0955 and 8.71% of the total variance.

A nominal logistic regression model with a dichotomous cataract variable as the response variable and species and radiation as predictors explained 21% of the variance (Table 2). There was an intermediate effect of radiation with an odds ratio of 0.284 (95% CI 0.186, 0.431), implying that the incidence of cataracts increased with level of background radiation. The odds ratio per unit change in the regressor was 0.722 (0.648, 0.804).

**Table 2:** Nominal logistic regression model of cataracts in relation to species and background radiation.

Variable	Chi-square	d.f.	*P*	Estimate (SE)	Odds ratio	95% CI for odds ratio
Species	182.12	56	<0.0001			
Radiation	98.29	1	0.0001	0.759 (0.084)	0.284	0.186, 0.431

*R* ^2^ was 0.21. The odds ratio and its 95% confidence interval is also reported.

There were weak, but significant phylogenetic signals in the cataracts data as evidenced by nested analyses. That was the case for genera nested within families (*F = *2,42 d.f. = 14, 22, *P* = 0.031) and for families nested within orders (*F = *56.17, d.f. = 16, 35, *P*<0.0001), but not for orders within super-orders (*F = *0.00, d.f. = 3, 51, *P* = 0.99).

**Figure 4:**
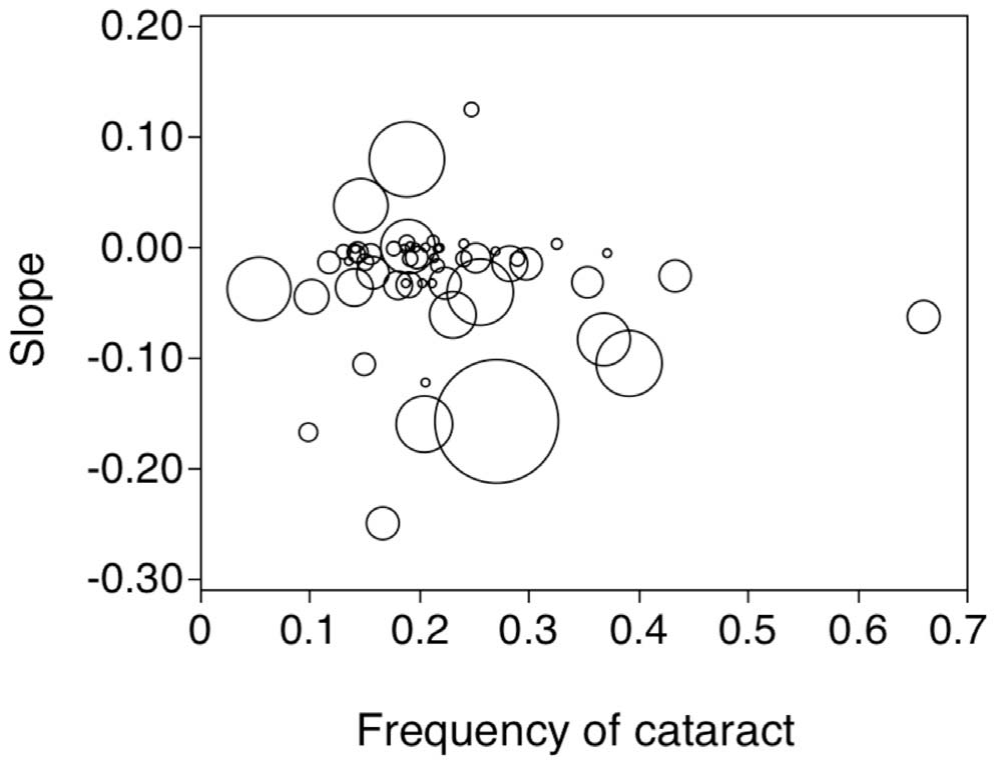
Relationship between slopes for the relationship between abundance and background radiation and the frequency of cataracts in different species of birds. The size of symbols reflects variation in sample size.

**Figure 5:**
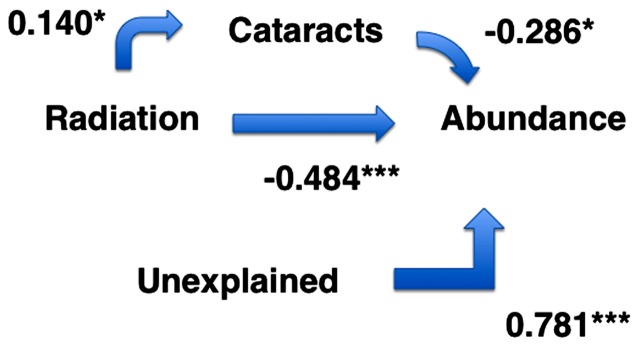
Path analysis of the direct and indirect relationships between cataracts and radiation, respectively, and abundance of breeding birds. *U* represents the unexplained variance. P-values for path coefficients are * *P*<0.05 and *** *P*<0.001.

The slope of the relationship between abundance and radiation was negatively related to the mean level of cataracts with an intermediate effect size (Figure 4; *F* = 4.91, d.f. = 1, 55, *r* ^2^ = 0.08, *P* = 0.031, estimate (SE) = −0.222 (0.100)). A path analysis showed that there was a weak positive effect of radiation on cataracts and a stronger negative effect on abundance of birds (Figure 5). In addition there was a negative effect of cataracts on abundance of birds (Figure 5). The indirect effect of radiation through cataracts on abundance was 0.140×0.286 = 0.040, while the direct effect was 0.484. While cataracts accounted for 4.0% (0.286×0.140) of the variance in abundance, radiation accounted for 23.4% (−0.484×−0.484). This implies that the effect of cataracts on abundance was a sixth of the effect of radiation *per se*. The unexplained variance was 61%.

## Discussion

The main findings of this study of cataracts in free-living birds in the Chernobyl region were that (1) the intensity and the incidence of cataracts increased with level of background radiation, (2) the slope of the relationship between abundance of breeding birds and background radiation decreased with increasing incidence of cataracts, but also through direct effects due to other diseases, food abundance or interspecific interactions in different species of birds, and (3) there was no increase in incidence of cataracts with increasing age.

Radiation cataracts are a frequent outcome of exposure to ionizing radiation [3]–[8]. While many studies have shown that atomic bomb survivors, workers in radiation facilities and people exposed to radiation following nuclear accidents suffer from elevated risk of developing cataracts [7], [11], [12], there are to the best of our knowledge no previous studies of animals. Cataracts in free-living birds in this study increased in a dose-dependent manner with level of background radiation with no evidence of a threshold. Individuals with opacities in one lens were much more likely to also develop opacities in the lens of the other eye, thereby increasing the risk of death for the individual. Here we have shown that radiation level in a mixed model accounted for an intermediate effect of 0.28, while a logistic regression based on dichotomous data showed an effect of a similar magnitude (0.30). The odds ratio for birds was 0.284 (95% CI 0.186, 0.431). This odds ratio is low compared to estimates for humans ranging from 1.25 (1.06–1.47) [12] over 1.30 (1.24–1.55) [11] to 1.54 (1.11–2.14) [7]. The odds ratio estimated for birds is bound to be conservative given the elevated risk of death experienced by individuals with cataracts. Although it may seem intuitively obvious that individual birds with cataracts suffer from reduced fitness, the magnitude of that cost has so far never been quantified in birds or other taxa.

Population size of birds can be assessed from point counts [22], [23] and mist net captures [39]. Birds and other organisms have severely depressed breeding population sizes in the most contaminated areas surrounding Chernobyl both in Ukraine and in Belarus [24], [26], [40]. Some of these effects may arise because of antioxidant deficiency [40], or because of excess mortality due to a variety of health reasons [41]. Studies of humans have shown that a great diversity of disease conditions has developed as a consequence of exposure to radiation from Chernobyl [8], [42]. If that was also the case in birds, as our data suggests, we should expect that the species with the strongest negative effects of radiation on abundance of breeding birds were the species with the highest frequency of disease. Thus we predicted a negative relationship between slope of the relationship between abundance and radiation and incidence of cataracts. This was indeed the relationship that we found. Obviously, we cannot ascertain that cataracts are the direct cause of these population declines, nor can we estimate the exact contribution of cataracts to this reduction. However, given that cataracts are associated with a dramatic increase in risk of mortality in birds, it seems likely that species with high frequencies and degree of cataracts will suffer from elevated levels of mortality. Furthermore, Galván et al. [18] have shown that bird species with pheomelanic coloration are more likely to develop cataracts than species without such coloration, and such species also show much stronger population declines from background radiation than expected [43], consistent with our predictions. We estimated the direct effects of background radiation, but also the indirect effects through cataracts, relying on path analysis (Figure 5). This resulted in an estimated indirect contribution of cataracts by 4.0% and a direct contribution of radiation by 23.4% to population levels. Therefore, radiation had a direct effect that was six-fold greater than the indirect effect of cataracts. However, again, we must emphasize that the indirect effect is bound to be an under-estimate given the likely elevated probability of mortality among individuals with cataracts. Possible direct effects of radiation on abundance include effects on other diseases [44], abundance of food [45] and interspecific interactions [46], [47].

Most human cases of cataracts are reported in people older than 40 years [3]–[8]. However, radiation cataracts can affect even young children, as shown in a study of people exposed to radiation from Chernobyl in Belarus [3] and studies of children treated with radiation therapy for cancer [48]–[50]. In addition, recent reports indicated that health care technicians who use radiation for medical treatment are at much higher risk of cataract irrespective of sex or age [51]. Here we were able to age more than half of all birds as either yearlings or older individuals using well-established external age criteria [20]. When entering age as a categorical predictor of cataracts in our statistical models together with the level of background radiation and species, we found only weak and non-significant effects of age on the incidence and the severity of cataracts. Hence radiation exposure rather than age was the main predictor of cataract. This conclusion must be drawn cautiously because high mortality rates among individuals with cataracts may have significantly reduced the fraction of old individuals that are more likely to develop cataracts.

In conclusion, we have shown increased incidence of cataracts in free-living birds inhabiting areas with elevated levels of background radiation around Chernobyl. Cataracts were associated with depressed breeding population sizes in contaminated areas.

## Supporting Information

Table S1 **Frequency of cataract, slope of the relationship between abundance and level of background radiation, and sample size.** See Methods for further details.
(DOC)
