# Supplementary material for: Elevated Frequency of Cataracts in Birds from Chernobyl
Source: PLoS One. 2013 Jul 30;8(7):e66939. doi: 10.1371/journal.pone.0066939 (PMC3728354; doi:10.1371/journal.pone.0066939)
Supplement: Table S1 — Frequency of cataract, slope of the relationship between abundance and level of background radiation, and sample size. See Methods for further details. (DOC) [file pone.0066939.s001.doc]

Table S1: Frequency of cataract, slope of the relationship between abundance and level of background radiation, and sample size. See Methods for further details.

| Species | Frequency of cataract | Slope | Sample size |
| --- | --- | --- | --- |
| *Acrocephalus palustris* | 0.155 | -0.0057 | 19 |
| *Acrocephalus scirpaceus* | 0.212 | 0.0060 | 6 |
| *Aegitahlos caudatus* | 0.188 | 0.0043 | 11 |
| *Anthus trivialis* | 0.230 | -0.0608 | 96 |
| *Caprimulgus europaeus* | 0.196 | 0.0002 | 1 |
| *Carduelis carduelis* | 0.190 | -0.0335 | 29 |
| *Certhia familiaris* | 0.205 | -0.1220 | 1 |
| *Coccothraustes coccothraustes* | 0.289 | -0.0101 | 9 |
| *Cuculus canorus* | 0.188 | 0.0801 | 251 |
| *Dendrocopos leucotos* | 0.218 | -0.0000 | 2 |
| *Dendrocopos major* | 0.223 | -0.0321 | 45 |
| *Dendrocopos medius* | 0.202 | -0.0321 | 1 |
| *Dendrocopos minor* | 0.187 | -0.0321 | 1 |
| *Dendrocopos syriacus* | 0.211 | -0.0321 | 1 |
| *Emberiza citrinella* | 0.204 | -0.1597 | 141 |
| *Emberiza schoeniclus* | 0.269 | -0.0031 | 1 |
| *Erithacus rubecula* | 0.282 | -0.0144 | 56 |
| *Ficedula hypoleuca* | 0.251 | -0.0092 | 39 |
| *Ficedula parva* | 0.213 | -0.0092 | 1 |
| *Fringilla coelebs* | 0.270 | -0.1572 | 676 |
| *Garrulus glandarius* | 0.247 | 0.1252 | 9 |
| *Hippolais icterina* | 0.157 | -0.0226 | 46 |
| *Hirundo rustica* | 0.660 | -0.0625 | 47 |
| *Jynx torquilla* | 0.143 | -0.0041 | 19 |
| *Lanius collurio* | 0.433 | -0.0255 | 45 |
| *Locustella fluviatilis* | 0.191 | 0.0009 | 4 |
| *Lullula arborea* | 0.180 | -0.0340 | 36 |
| *Luscinia luscinia* | 0.353 | -0.0312 | 43 |
| *Motacilla alba* | 0.098 | -0.1670 | 15 |
| *Motacilla flava* | 0.216 | -0.0006 | 1 |
| *Muscicapa striata* | 0.150 | -0.0130 | 12 |
| *Oriolus oriolus* | 0.189 | 0.0010 | 131 |
| *Parus ater* | 0.297 | -0.0145 | 46 |
| *Parus caeruleus* | 0.140 | -0.0360 | 62 |
| *Parus cristatus* | 0.130 | -0.0036 | 9 |
| *Parus major* | 0.255 | -0.0402 | 195 |
| *Parus montanus* | 0.142 | -0.0053 | 13 |
| *Parus palustris* | 0.240 | 0.0034 | 4 |
| *Phoenicurus ochruros* | 0.371 | -0.0049 | 3 |
| *Phylloscopus collybita* | 0.101 | -0.0443 | 53 |
| *Phylloscopus sibilatrix* | 0.053 | -0.0373 | 179 |
| *Phylloscopus trochilus* | 0.146 | 0.0381 | 130 |
| *Picus canus* | 0.205 | 0.0003 | 1 |
| *Pyrrhuls pyrrhula* | 0.186 | -0.0008 | 1 |
| *Sitta europaea* | 0.216 | -0.0162 | 8 |
| *Sturnus vulgaris* | 0.117 | -0.0133 | 22 |
| *Sylvia atricapilla* | 0.149 | -0.1055 | 22 |
| *Sylvia borin* | 0.197 | -0.0088 | 22 |
| *Sylvia communis* | 0.135 | -0.0120 | 3 |
| *Sylvia curruca* | 0.176 | -0.0008 | 9 |
| *Sylvia nisoria* | 0.141 | -0.0009 | 2 |
| *Troglodytes troglodytes* | 0.191 | -0.0099 | 10 |
| *Turdus merula* | 0.391 | -0.1047 | 192 |
| *Turdus philomelos* | 0.368 | -0.0830 | 123 |
| *Turdus pilaris* | 0.240 | -0.0099 | 11 |
| *Turdus viscivorus* | 0.325 | 0.0035 | 5 |
| *Upupa epops* | 0.166 | -0.2495 | 47 |
